# Comparative Study of Machine Learning Models for Optimal Prediction of Printed-Line Features in Material Extrusion Additive Manufacturing

**DOI:** 10.3390/ma19143092

**Published:** 2026-07-17

**Authors:** Shuhao Shen, Ruohan Chen, Wenjie Sun, Meiya Zhao, Haining Zhang

**Affiliations:** 1 School of Information Engineering, Suzhou University, Suzhou 234000, China; shenshuhao@ahszu.edu.cn (S.S.); crh@stu.ahszu.edu.cn (R.C.); swj@stu.ahszu.edu.cn (W.S.); zmy@stu.ahszu.edu.cn (M.Z.); 2 KAIST InnoCORE PRISM-AI Center, Korea Advanced Institute of Science and Technology (KAIST), Daejeon 34141, Republic of Korea

**Keywords:** material extrusion, additive manufacturing, fused deposition modeling, machine learning, process optimization, edge non-uniformity, Gaussian process regression, explainable artificial intelligence

## Abstract

Material extrusion (MEX), commonly known as fused deposition modeling (FDM), has become a widely adopted additive manufacturing (AM) technology owing to its low equipment cost and broad polymer compatibility. However, the geometric fidelity of the printed line often suffers from defects that compromise overall part quality. Specifically, residual edge non-uniformity degrades surface finish, while uncontrolled line width variability causes undesired gaps or overlaps that undermine mechanical performance. Therefore, ensuring an accurate line width and low edge non-uniformity is essential for advancing material extrusion toward high-precision industrial applications. In this study, a machine learning framework is proposed for the rapid prediction and analysis of printed line characteristics. Nozzle temperature, print speed, and material flow rate were considered as input process parameters. Mean line width and edge non-uniformity were taken as the target responses. Four representative machine learning algorithms (XGBoost, BPNN, GPR, and SVR) were adopted for model development. To enhance predictive accuracy, these models were optimized using Particle Swarm Optimization for automatic hyperparameter tuning. Subsequently, comparative evaluations identified GPR as the optimal predictive model. Furthermore, a SHAP-based interpretability analysis was conducted, revealing that nozzle temperature dominates line width, while the flow rate governs edge non-uniformity. Consequently, this interpretable and computationally efficient surrogate modeling approach provides a robust foundation for future closed-loop quality control and inverse process design.

## 1. Introduction

With the increasing adoption of additive manufacturing (AM) in low-volume customized production and on-demand end-use part fabrication, material extrusion (MEX), commonly known as fused deposition modeling (FDM) and fused filament fabrication (FFF), has become one of the most widely adopted AM technologies, owing to its low equipment cost, broad polymer compatibility, and considerable design freedom [1,2,3]. Specifically, MEX has now been successfully deployed across diverse industries, including biomedical scaffolds [4,5], lightweight aerospace components [6,7], conformal electronics packaging [8], and personalized consumer products [9].

Although MEX offers many advantages, the geometric fidelity of its basic deposition unit, the printed line, has emerged as a critical determinant of overall part quality, as these lines often suffer from defects that compromise mechanical integrity, dimensional accuracy, and surface finish [10,11]. Within an inappropriate process window, the printed lines may exhibit a wide spectrum of defects such as discontinuous strands, excessive material spreading, rough surface textures, and non-uniform distribution [12,13,14]. Most of these gross defects can largely be eliminated by experimentally identifying a “normal” MEX working window through process screening. Nevertheless, even within such an optimized working window, the printed lines still exhibit non-trivial edge non-uniformity and inevitable line-width variability. The residual edge non-uniformity directly degrades the surface quality of the final part, while uncontrolled width variability undermines the precision of densely packed toolpaths, causing undesired inter-line gaps or overlaps that compromise mechanical performance and resolution [15,16]. Hence, ensuring low edge non-uniformity and accurate, predictable line width within the MEX working window has become a central concern for further advancing MEX toward high-precision industrial applications.

At present, the relationship between MEX process parameters and the resulting line features is most commonly examined through trial-and-error experimentation or response-surface methodology (RSM) based on design-of-experiments (DOE) [17,18]. While RSM provides closed-form polynomial surrogates, the fitted surfaces are constrained to low-order curvature and tend to misrepresent the strongly nonlinear couplings between process inputs and line responses, limiting their predictive accuracy across the working space. To obtain mechanistic insight, computational fluid dynamics (CFD) and thermo-mechanical finite-element models have also been developed to simulate filament melting, extrusion, deposition, and solidification [19,20,21]. Although such physics-based models reveal the underlying mechanisms, their high computational cost and reliance on accurate constitutive data make them impractical for rapid, real-time prediction during process planning.

Machine learning (ML)-based surrogate modeling has recently emerged as a powerful complementary framework that overcomes the accuracy, efficiency, and interpretability trade-offs of conventional approaches [22,23]. By learning the input–output mapping directly from experimental data, ML models can deliver near-instant predictions once trained, require no detailed physical assumptions, and naturally accommodate complex nonlinear interactions among process parameters [24,25,26]. Various ML techniques have been applied across AM for response prediction and quality monitoring [27,28,29,30]. However, a fair and systematic comparison of representative ML model families on the specific task of MEX line-feature prediction, together with rigorous hyperparameter optimization and post hoc interpretability analysis, remains scarce.

To address this gap, the present study systematically investigates and compares several representative ML methods for the rapid prediction of printed-line width and edge non-uniformity in MEX. Three primary process parameters are considered as model inputs: nozzle temperature, print speed, and material flow rate. Rigorous hyperparameter optimization is performed to ensure each model operates at its full predictive potential, and a post hoc interpretability analysis is conducted to dissect the contribution of each process parameter to the predicted-line responses. The present work focuses on establishing an accurate, interpretable, and computationally efficient surrogate modeling framework that captures the residual line-feature variability within the MEX working window, providing a foundation for future closed-loop quality control and inverse process design. For consistency with international standards, this work uses material extrusion (MEX) following ISO/ASTM 52900 [31], while recognizing that the process is also commonly referred to as FDM or FFF, based on the foundational work of Crump [32]. The artificial intelligence terminology follows ISO/IEC 22989 [33], and edge non-uniformity is defined with reference to the surface-texture terminology in ISO 21920-2 [34].

The remainder of this paper proceeds as follows. Section 2 and Section 3 comprehensively detail the experimental design and the employed machine learning methodologies, respectively. The subsequent modeling and optimization outcomes are evaluated in Section 4, followed by concluding remarks and future perspectives in Section 5.

## 2. Experimental Setup and Feature Analysis

### 2.1. Working Principles and Experimental Design

**Figure 1:**
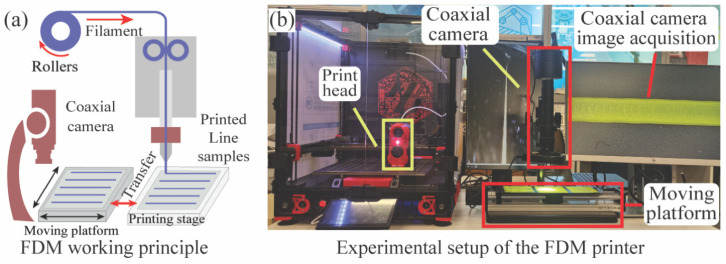
Experimental setup and working principle of the MEX 3D printer: (**a**) schematic of the FDM working principle, (**b**) photograph of the experimental setup with the coaxial camera for real-time printed-line image acquisition.

As shown in Figure 1, an open-source Voron MEX printer (CX Technology, Shenzhen, China) was deployed for the experimental investigation, selected for its cost-efficiency alongside built-in Z-probing and bootloader functionalities. 

Real-time bidirectional communication was established via a USB-CDC protocol and governed by a Raspberry Pi 4 Model B gateway (Raspberry Pi Ltd., Cambridge, UK). Utilizing this integrated hardware platform, a Latin Hypercube Sampling (LHS) method was implemented to structure the experimental design [35,36]. Print speed, flow rate multiplier, and nozzle temperature were defined as independent parameters to evaluate their impact on primary responses, specifically the width and edge non-uniformity of the printed tracks. In terms of hardware, the open-source Voron platform is a direct-drive material-extrusion system fitted with a 0.4 mm nozzle and fed with standard 1.75 mm ABS filament (Table 1); the print-speed and flow-rate ranges investigated were kept within the maximum volumetric throughput of the nozzle–extruder assembly, so as to minimize the possibility that insufficient filament feeding affected the measured deposition defects. 

**Figure 2:**
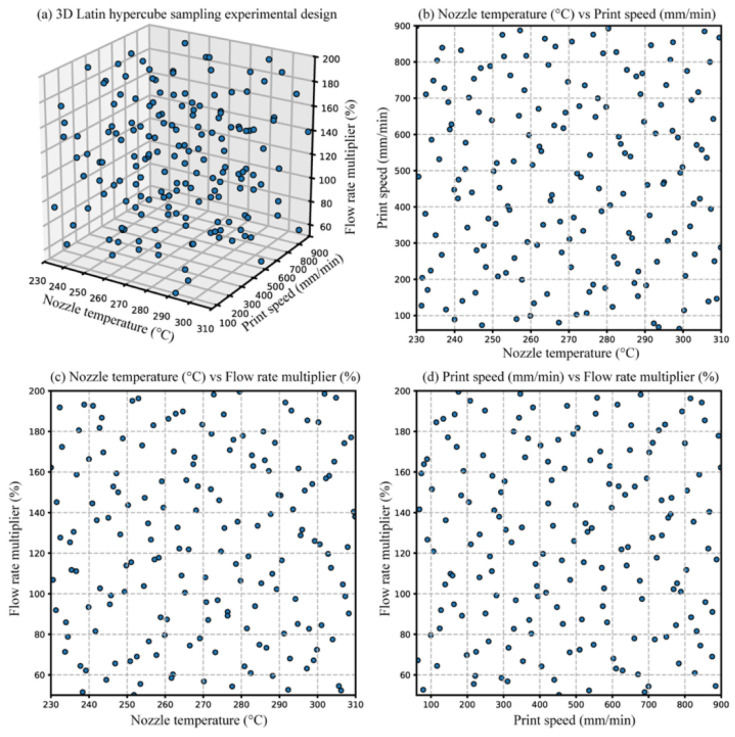
Distribution of the experimental design space generated via Latin Hypercube Sampling (LHS), visualized in a 3D scatter plot and corresponding 2D projections to ensure comprehensive spatial coverage.

The operational ranges for these parameters, summarized in Table 1, were set as follows: nozzle temperature from 230 °C to 310 °C, print speed from 60 mm/min to 900 mm/min, and flow rate multiplier from 50% to 200%. To isolate the effects of these variables, all other processing factors were held constant. To sufficiently cover this three-dimensional design space, 169 experimental points were generated via the LHS strategy. This approach partitioned each parameter range into 169 mutually exclusive, equiprobable intervals, thereby maximizing the space-filling uniformity of the dataset. This distribution is visualized in Figure 2, where the 3D scatter plot (Figure 2a) and the corresponding 2D projections (Figure 2b–d) confirm the absence of clustering and ensure comprehensive coverage of the design space. Such a configuration is essential for capturing both dominant single-parameter trends and complex higher-order interactions while maintaining experimental tractability. For physical execution, a 50 mm single-track filament was extruded for each parametric combination, with each trial performed in triplicate to suppress stochastic noise, resulting in a total of 507 deposited tracks for subsequent morphological extraction. It should be noted that these ranges intentionally extend beyond the manufacturer’s nominal ABS processing window in order to deliberately elicit and characterize the deposition defects (excessive lateral spreading, edge instability, and non-uniform deposition) that the present models are designed to predict; confining the design space to the datasheet window would exclude precisely these phenomena. Moreover, the tabulated nozzle temperature denotes the hot-end set-point rather than the actual polymer melt temperature, which is lower owing to the finite residence time and convective losses within the nozzle. Here, the 100 °C build-plate temperature is consistent with the recommended processing window for ABS (typically 90–110 °C), and the print speed is expressed in mm/min (60–900 mm/min, i.e., approximately 1–15 mm/s). The nozzle-temperature range spans the normal ABS window (about 230–270 °C) and is intentionally extended to 310 °C to elicit the over-temperature deposition defects targeted by the models.

### 2.2. Feature Extraction

**Figure 3:**
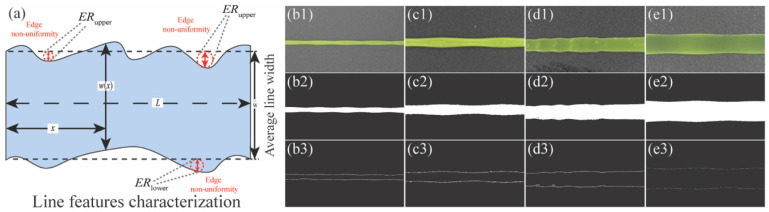
Image processing for printed-line feature characterization: (**a**) definitions of average line width and edge non-uniformity, (**b1**–**e1**) original optical microscope images, (**b2**–**e2**) segmented binary masks, and (**b3**–**e3**) extracted-line edge boundaries.

The printed-line features, including line width and line edge non-uniformity, are defined in Figure 3a. The morphological characterization of the deposited tracks was carried out through a sequential image processing pipeline. First, the original images of the printed-line samples were acquired by an optical microscope under consistent illumination and magnification conditions (Figure 3(b1–e1)). Since the raw images inevitably contain background interference, uneven illumination, and minor surface texture variations that are not representative of the actual track geometry, an image processing algorithm was subsequently applied to segment the main body of the filament from the substrate. As illustrated in Figure 3(b2–e2), the segmented binary masks of the deposited tracks were obtained through grayscale conversion, adaptive thresholding, and morphological operations, effectively isolating the printed-line region from the background. Building upon the segmented track bodies, the upper and lower edges of each printed line were then extracted via boundary detection, as shown in Figure 3(b3–e3), providing a discretized representation of the track contour suitable for quantitative analysis. Specifically, the printed-line images were captured with a coaxial digital microscope camera (Jingtuo Youcheng Technology, Shenzhen, China) at a resolution of 1024 × 1024 pixels and magnifications up to 40×.

With the upper and lower contours of each track extracted, two quantitative descriptors were derived to characterize the track geometry. The track was sampled column-by-column along its longitudinal direction, and at every column *i*, the local width *w_i_* was obtained as the vertical distance between the two opposing edge points. Averaging these column-wise measurements yielded the mean track width:

(1) $$\bar{w}=\frac{1}{N}\sum_{i=1}^{N}w_{i}$$

with N denoting the number of columns sampled across the entire track length.

To describe how the actual contour fluctuates around its ideal geometry, a reference baseline was first established for each side of the track by fitting a straight line through the corresponding edge points; the perpendicular offset of each edge point from this baseline was then taken as the local roughness contribution. The two sides were combined into a single scalar indicator, the mean edge non-uniformity, expressed as the root-mean-square of the upper- and lower-side deviations: This edge non-uniformity metric quantifies the waviness (nonlinearity) of the deposited-line edge rather than an areal surface roughness.

(2) $${ER}_{m}=\sqrt{\frac{1}{N}\sum_{i=1}^{N}\left({ER}_{upper,i}^{2}+{ER}_{lower,i}^{2}\right)}$$

In Equation (2), ${ER}_{upper,i}$ and ${ER}_{lower,i}$ correspond to the signed deviations of the *i*-th upper and lower edge points from their respective baselines. Together, $\bar{w}$ and ${ER}_{m}$ constitute a compact two-dimensional feature vector that captures, respectively, the average dimensional fidelity and the boundary regularity of the deposited filament, and serve as the response variables in the subsequent modeling stage.

### 2.3. Statistical Feature Analysis of the Dataset 

**Figure 4:**
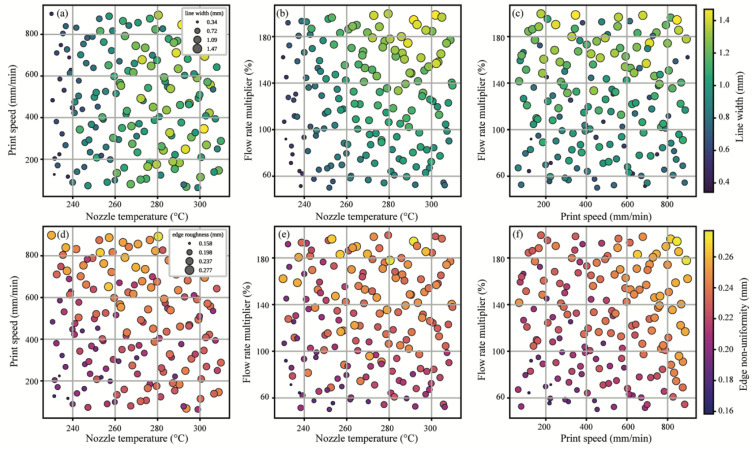
Bubble charts visualizing the relationships between MEX process parameters and printed-line responses: (**a**–**c**) line width and (**d**–**f**) edge non-uniformity.

Figure 4 visualizes the experimental dataset in a bubble chart format, in which the three process parameters are mapped onto the horizontal and vertical axes pairwise, while the two response variables are simultaneously encoded by both the diameter and the color of each bubble. Figure 4a–c and Figure 4d–f depict the mean line width *w_i_* and the mean edge non-uniformity ${ER}_{m}$, respectively. In every subplot, a larger and brighter (yellow-shifted) marker indicates a higher response value, whereas smaller and darker (purple-shifted) markers correspond to lower values. This dual encoding allows the dominant trends and the secondary parameter interactions to be inspected in a single view.

With a focus first on the line width (Figure 4a–c), a prominent gradient is observed along the nozzle temperature axis due to reduced melt viscosity and subsequent lateral spreading, complemented by a milder positive trend along the flow rate multiplier axis. Conversely, variations in print speed exert a negligible effect on the deposited volume. For edge non-uniformity (Figure 4d–f), a distinct pattern emerges: the highest values concentrate where both the print speed and flow rate multiplier are simultaneously elevated (upper-right corners of Figure 4e,f). This reveals that rapid kinematics combined with excessive material delivery promotes melt instability and irregular wetting at the track boundary, whereas balanced speed–flow combinations minimize roughness.

**Figure 5:**
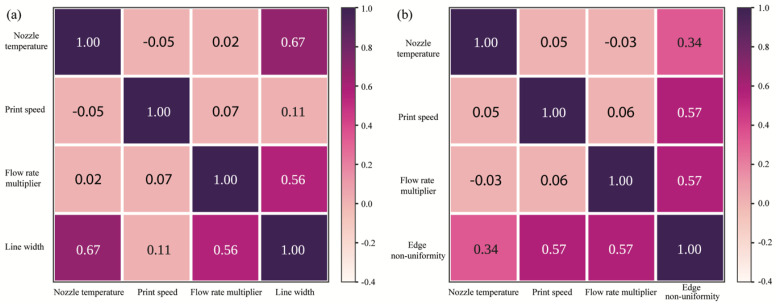
Pearson correlation coefficient heatmaps quantifying the linear relationships between the process parameters and the responses: (**a**) line width and (**b**) edge non-uniformity.

To quantify these observations, Pearson correlation coefficients were computed and summarized as heatmaps in Figure 5. Line width correlates strongly with nozzle temperature (R = 0.67) and flow rate multiplier (R = 0.56), but minimally with print speed (R = 0.11). In contrast, edge non-uniformity is dominated by the print speed and flow rate (R = 0.57 for both), clearly outpointing temperature (R = 0.34). This shift demonstrates that dimensional accuracy is governed primarily by thermal and volumetric melt conditions, whereas boundary regularity is highly sensitive to kinematic and feeding dynamics. As no single parameter controls both metrics uniformly, a multi-input, multi-output modeling strategy is justified to optimize overall track quality.

## 3. Methodology

**Figure 6:**
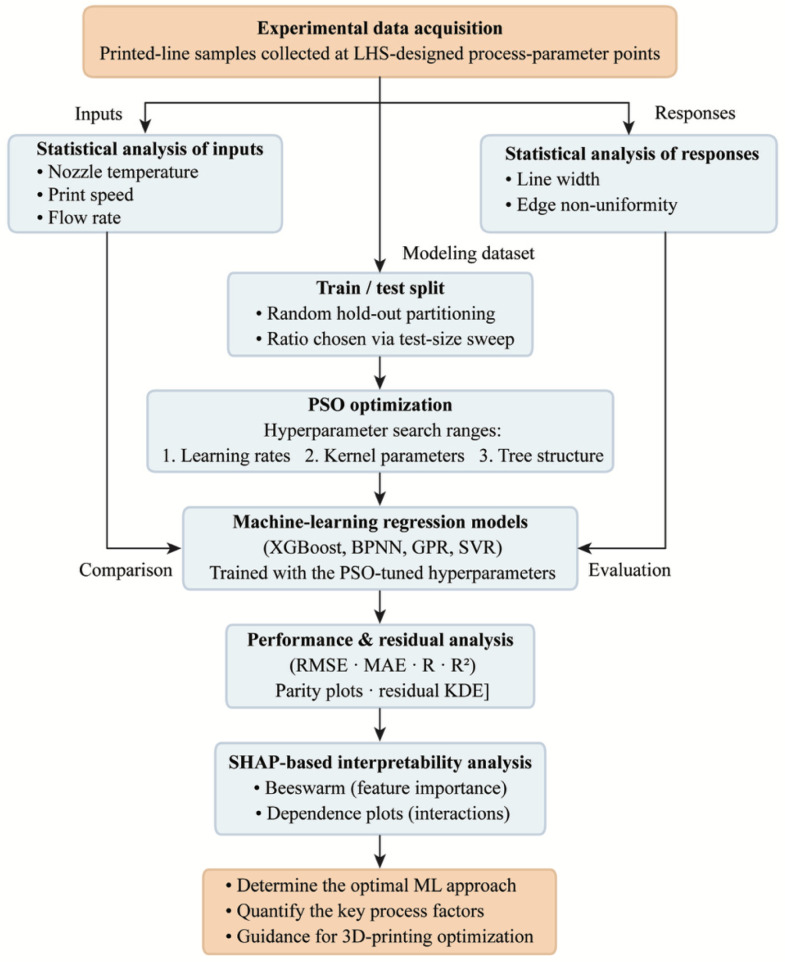
The proposed integrated machine learning framework for the prediction, optimization, and interpretability analysis of printed-line features.

As shown in Figure 6, this study proposes an integrated, comparative machine learning framework for predicting and optimizing printed-line features in MEX 3D printing. Specifically, printed-line samples are first collected at process-parameter points designed by Latin Hypercube Sampling, after which the inputs (nozzle temperature, print speed, and flow rate) and the responses (line width and edge non-uniformity) are each characterized through statistical analysis. The resulting modeling dataset is divided via random hold-out partitioning, with the split ratio chosen through a test-size sweep, and Particle Swarm Optimization is used to tune model hyperparameters across learning rates, kernel parameters, and tree structures. With these optimized settings, four representative regression models are trained and comparatively evaluated through performance and residual analysis (RMSE, MAE, R, and R^2^, together with parity plots and residual KDE distributions), while a SHAP-based interpretability analysis uses beeswarm and dependence plots to rank feature importance and reveal parameter interactions. Through this comparison, the framework identifies the best-performing model for optimal prediction of printed-line features, quantifies the key governing process factors, and provides practical guidance for MEX 3D-printing optimization.

### 3.1. Modeling Methods

**Figure 7:**
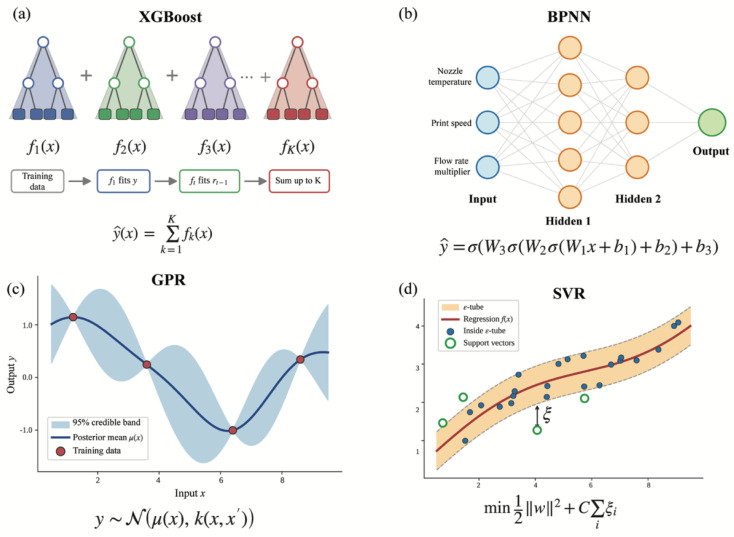
Schematic representations of the four adopted machine learning regression algorithms: (**a**) XGBoost, (**b**) BPNN, (**c**) GPR, and (**d**) SVR.

To enable a comprehensive comparison, four regression models spanning distinct learning paradigms are adopted, namely gradient-boosted trees (XGBoost), a neural network (BPNN), a probabilistic kernel method (GPR), and a margin-based kernel method (SVR), as illustrated in Figure 7. Because each represents the relationship between process parameters and printed-line features in a fundamentally different way, that is, in piecewise, continuous, probabilistic, and margin-based forms, together they provide a broad basis for evaluating which modeling strategy is more suited to the present problem. These four algorithms were selected because they are the most widely reported and best-performing regressors for additive-manufacturing property prediction under the small, noisy, and strongly nonlinear datasets typical of process-parameter studies, where deep architectures that require large samples are prone to overfitting [37]. Together, they span the four dominant supervised-learning paradigms, namely ensemble trees, neural networks, probabilistic kernels, and margin-based kernels, so that the comparison is not biased toward a single inductive assumption and the modeling strategy most appropriate for MEX line-feature prediction can be identified objectively rather than presumed. Simpler linear or low-order polynomial surrogates such as RSM were excluded because they cannot capture the strong nonlinear couplings among the process parameters, whereas data-intensive deep networks were considered unsuitable for the present sample size.

XGBoost (Figure 7a) is an additive ensemble of gradient-boosted regression trees in which each successive tree $f_{k}(x)$ is fitted to the residuals of the previous ensemble and the predictions are summed as $\hat{y}(x)=\sum_{k=1}^{K}f_{k}(x)$, repeatedly partitioning the parameter space to capture sharp nonlinear effects while regularization limits overfitting [38,39]. Since this mapping remains piecewise-constant, the BPNN (Figure 7b), a feed-forward network with two hidden layers expressed as $\hat{y}=\sigma \;(W_{3}\sigma (W_{2}\sigma (W_{1}x+b_{1})+b_{2})+b_{3})$, instead composes successive nonlinear transformations and adjusts its weights and biases through back-propagation to approximate smooth continuous responses [40]. Both models, however, return only point estimates, whereas GPR (Figure 7c) treats the output as a Gaussian process, y∼N(μ(x),k(x,x′)), yielding not only a posterior-mean prediction but also the credible interval shown by the shaded band in Figure 7c, which quantifies predictive uncertainty under limited data [41,42]. Finally, SVR (Figure 7d) fits a regression function within an ε-insensitive tube by solving $\min1/2∥w{∥}^{2}+C\sum_{i}\xi_{i}$, so that only the support vectors define the model; when combined with the kernel trick, this margin-based formulation tolerates small deviations and yields robust generalization under limited and noisy data [43,44]. Together, these four models constitute the diverse basis on which the comparative evaluation in the following sections is built.

The schematics in Figure 7 make these differences explicit. Panel (a) depicts XGBoost as an additive ensemble in which shallow regression trees are appended sequentially, each correcting the residuals of the preceding ones. Panel (b) shows the BPNN as a fully connected feed-forward network that propagates the three process inputs through two hidden layers to the predicted response. Panel (c) illustrates GPR, in which the shaded band represents the posterior predictive distribution together with its confidence interval, thereby conveying predictive uncertainty. Panel (d) represents SVR, in which an ε-insensitive tube around the regression function is supported by the identified support vectors. Together, the four panels clarify how each paradigm maps the MEX process parameters to the printed-line responses.

### 3.2. Selection of Model Hyperparameters

**Figure 8:**
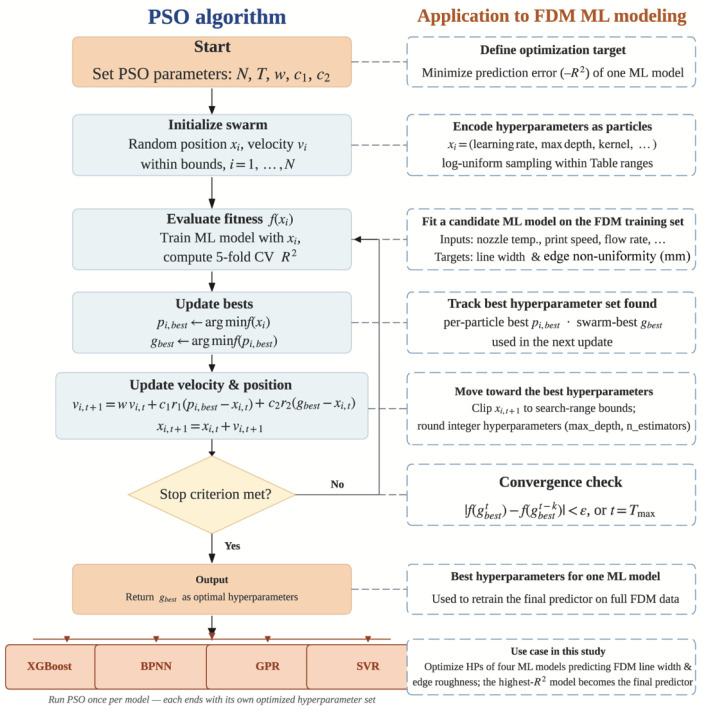
Flowchart of the Particle Swarm Optimization (PSO) algorithm applied for automated hyperparameter tuning of the machine learning models.

The predictive accuracy of each model depends strongly on its hyperparameters, yet the relevant search space is high-dimensional, continuous, and spans several orders of magnitude, which makes manual or grid search inefficient. Particle Swarm Optimization (PSO), a gradient-free global metaheuristic, is therefore employed to tune all four models automatically, following the procedure shown in Figure 8. Compared with manual, grid, or random search, population-based metaheuristics such as PSO explore the hyperparameter space more efficiently and have been shown to locate near-optimal configurations at a lower computational cost, particularly for the high-dimensional, non-convex search spaces of tree ensembles, neural networks, and kernel methods [37,45]. PSO is adopted here for its few control parameters, fast convergence, and repeated success in tuning machine learning models across engineering domains [45].

In PSO, each candidate hyperparameter set is encoded as a particle whose position $x_{i}$ moves through the search space according to its own best solution $p_{best}$ and the swarm best $g_{best}$:

(3) $$v_{i,t+1}=w\;v_{i,t}+c_{1}r_{1}(p_{best}-x_{i,t})+c_{2}r_{2}(g_{best}-x_{i,t}),\;\;x_{i,t+1}=x_{i,t}+v_{i,t+1}$$

where w is the inertia weight, $c_{1}$ and $c_{2}$ the cognitive and social factors, and $r_{1},r_{2}$ random numbers in [0, 1]. The swarm is iterated until the change in $g_{best}$ falls below a tolerance ε or the maximum number of iterations $T_{max}$ is reached. The optimization target is to minimize the prediction error, expressed as the fitness $-R^{2}$, which for each candidate particle is evaluated by training the model on the training set with five-fold cross-validation, thereby guarding against overfitting.

The hyperparameters optimized for each model and their search ranges are summarized in Table 2. These include the learning rate, number of estimators, and maximum depth for XGBoost; the L2 regularization strength and the two hidden-layer sizes for BPNN; the length scale and noise variance for GPR; and the penalty C, kernel coefficient γ, and tube width ε for SVR. Continuous parameters spanning wide ranges are sampled on a logarithmic scale, while integer parameters such as maximum depth and the number of estimators are rounded after each update. Specifically, for XGBoost, learning_rate scales the contribution of each successive tree (shrinkage), n_estimators sets the number of boosting rounds, and max_depth limits the depth and hence the complexity of each tree. For BPNN, alpha is the L2 regularization coefficient that penalizes large weights to mitigate overfitting, while hidden_size_1 and hidden_size_2 define the number of neurons in the first and second hidden layers (hidden_size_2 = 0 denotes a single-hidden-layer network). For GPR, the length scale ℓ of the radial-basis-function kernel controls the smoothness and correlation range of the fitted function, and the noise variance σ_n_^2^ accounts for observation noise added to the kernel diagonal. For SVR with an RBF kernel, the penalty C balances model flatness against tolerance to training errors, the kernel coefficient γ sets the influence radius of a single training sample, and the tube width ε defines the ε-insensitive zone within which deviations are not penalized. All hyperparameter designations used in the text correspond exactly to those listed in Table 2.

PSO is run once per model, so that each obtains its own optimized hyperparameter set, which is then used to retrain the predictor on the full MEX dataset. The resulting tuned models provide the basis for the comparative performance evaluation presented in the following section, in which the model achieving the highest $R^{2}$ is selected as the final predictor.

### 3.3. Dataset Pre-Processing and Model Evaluation

As the printed-line features are recorded in different units and on different dimensional scales, directly feeding the raw values into the models would undermine their comparability and degrade fitting performance. A further difficulty arises from the strongly unequal variance between the process parameters and the printed-line responses: indicators carrying disproportionately large variance tend to dominate the learning process and bias the resulting model. To eliminate both effects, every variable in the dataset was standardized prior to modeling, and the outliers detected during this step were removed.

The cleaned and standardized dataset was then partitioned into a training subset and a testing subset, the former used to fit the models and the latter reserved for assessing their predictive ability on unseen samples. Since this partition can markedly affect the outcome, the proportion allocated to the test subset was swept from 10% to 45% in increments of 5%, and the correlation coefficient R was used to identify the most suitable split. Once the models had been trained, four widely used metrics, namely the mean absolute error (MAE), the root-mean-square error (RMSE), the correlation coefficient R, and the coefficient of determination $R^{2}$, were computed to compare the candidates and select the best-performing model. All models were implemented in Python 3.10 using the open-source libraries scikit-learn 1.3.2 (for BPNN, GPR, and SVR), XGBoost 2.0.3 (for XGBoost), PySwarms 1.3.0 (for the Particle Swarm Optimization routine), and SHAP 0.44.1 (for the interpretability analysis) [46,47]. To ensure reproducibility, a fixed random seed (random_state = 42) was assumed for the final train–test split and for model training, so that the reported results can be exactly reproduced; for the test-size sensitivity study in Section 4.1, the split was instead repeated over multiple random seeds and averaged to remove the influence of any single partition.

(4) $$RMSE=\frac{1}{n}\sqrt{\sum_{i=1}^{n}(f(x_{i})-y_{i}{)}^{2}}$$

(5) $$MAE=\frac{1}{n}\sum_{i=1}^{n}|f(x_{i})-y_{i}|$$

(6) $$R=\frac{\sum_{i=1}^{n}(f(x_{i})-\bar{f(x_{i})})(y_{i}-\bar{y})}{\sqrt{\sum_{i=1}^{n}(f(x_{i})-\bar{f(x_{i})}{)}^{2}}\;\sqrt{\sum_{i=1}^{n}(y_{i}-\bar{y}{)}^{2}}}$$

(7) $$R^{2}=1-\frac{\sum_{i=1}^{n}(y_{i}-f(x_{i}){)}^{2}}{\sum_{i=1}^{n}(y_{i}-\bar{y}{)}^{2}}$$

where $y_{i}$ and $\bar{y}$ denote the measured value and its mean, n is the number of samples, and $f(x_{i})$ is the model prediction with mean $\bar{f(x_{i})}$.

### 3.4. SHAP-Based Interpretability Analysis

To interpret the trained models and quantify the contribution of each process parameter to the predicted-line features, a post hoc SHapley Additive exPlanations (SHAP) analysis was performed. SHAP, a model-agnostic method grounded in cooperative game theory, quantifies the positive or negative contribution of each feature to the target and is widely used for model interpretation [48,49]. For a given prediction *f*(*x*), SHAP assigns to each feature *i* a Shapley value φ_i_ equal to the average marginal contribution of that feature over all possible feature subsets *S*:

*φ_i_* = Σ (|*S*|! (|*F*| − |*S*| − *1*)!/|*F*|!) · [*f*(*S* ∪ {*i*}) − *f*(*S*)], *S* ⊆ *F*\{*i*} (8)

where F is the full set of input features. The model output is thereby decomposed additively as *f*(*x*) = *φ*_0_ + Σ*_i_ φ_i_*, in which *φ*_0_ is the expected (baseline) model output. A positive *φ_i_* indicates that the feature pushes the prediction above the baseline, whereas a negative value pulls it below. The resulting Shapley values are aggregated into beeswarm plots to rank global feature importance and into dependence plots to reveal how each parameter influences the response across its range. The corresponding results are presented and discussed in Section 4.3.

## 4. Results and Discussion

### 4.1. Determination of Dataset Division Ratio

**Figure 9:**
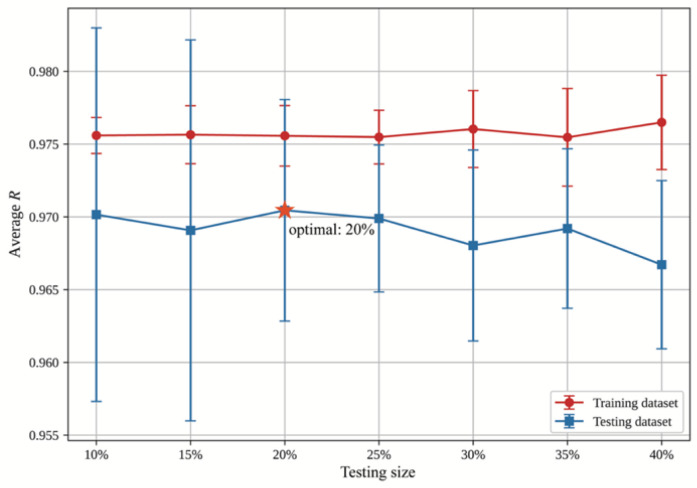
Effect of the testing dataset size proportion on the average correlation coefficient (R) for the GPR model to determine the optimal dataset division ratio.

Since the partition ratio can considerably affect model performance, the size of the testing subset was varied from 10% to 40% in steps of 5%. To suppress the randomness inherent in a single run, the modeling was repeated 60 times for every ratio, and the mean correlation coefficient R was taken as the representative measure of performance under that ratio. Figure 9 illustrates this influence using the GPR model as a representative case. For the training subset, the partition exerted only a minor effect, with R remaining close to 0.975 across the entire range. The testing subset yielded consistently lower values that varied around 0.97, reflecting the expected gap between in-sample fitting and out-of-sample prediction. As the testing proportion grew from 10% to 20%, the average R rose to its maximum, after which it gradually declined as the ratio was further enlarged toward 40%. Because the GPR model attained its peak accuracy at a testing proportion of 20%, and the XGBoost, BPNN, and SVR models exhibited the same tendency, a training-to-testing split of 80%:20% was adopted as the optimal division ratio in this study. This empirically identified 80:20 partition is also consistent with the ratio most commonly adopted in the machine learning literature, where allocating 70–80% of the data to training and 20–30% to testing has been shown, both empirically and theoretically, to balance estimation bias against evaluation variance [50,51].

### 4.2. Comparative Analysis of Modeling Performance

**Figure 10:**
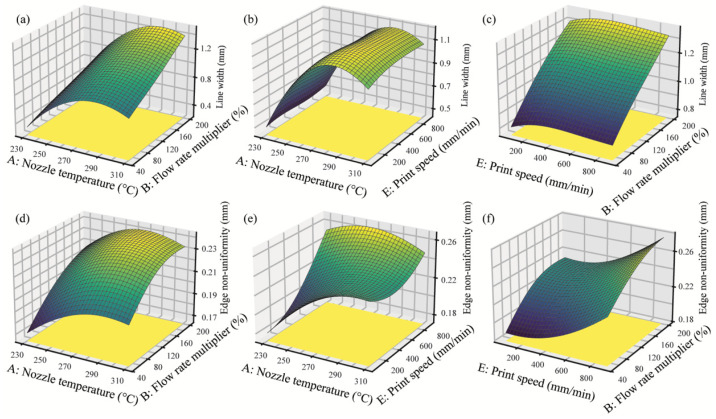
Response surface plots illustrating the combined effects of process parameters on (**a**–**c**) line width and (**d**–**f**) edge non-uniformity.

Figure 10 illustrates the overall cause-and-effect relationships between the three process parameters (nozzle temperature, flow-rate multiplier, and print speed) and the two printed-line features. As shown in Figure 10a–c for the line width, the flow-rate multiplier exerts the most pronounced positive influence, since a larger volume of extruded material per unit length broadens the deposited line. The nozzle temperature is also positively correlated with the line width, because a higher temperature reduces the melt viscosity and promotes lateral spreading of the filament. By comparison, the print speed plays a relatively minor role, and a faster traverse tends to narrow the line as less material is deposited per unit length. For the line non-uniformity, presented in Figure 10d–f, both the nozzle temperature and the flow-rate multiplier show a positive relationship, as excessive heat and over-extrusion intensify local material accumulation and edge irregularity. The print speed further increases the roughness at high values due to unstable deposition. Accordingly, a wide yet smooth line is mainly governed by the combined effect of flow-rate multiplier and nozzle temperature, and the lowest edge non-uniformity is achieved under a moderate temperature together with a balanced flow-rate setting.

**Figure 11:**
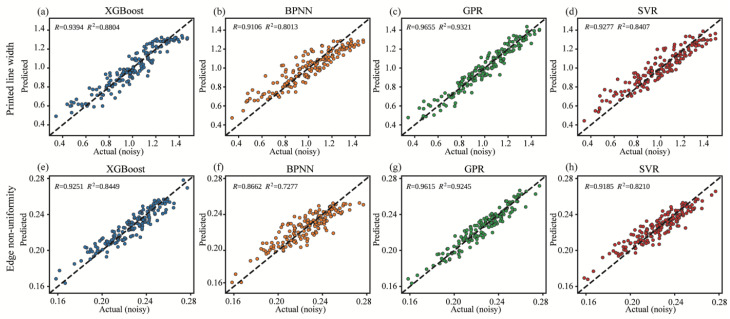
Parity plots comparing the actual and predicted values for the four optimized machine learning models: (**a**–**d**) line width and (**e**–**h**) edge non-uniformity.

To identify the most suitable model for accurately predicting the printed-line features in MEX, the modeling performance of the four candidate models based on XGBoost, BPNN, GPR, and SVR was compared as follows. As shown in Figure 11, R and R^2^ were first adopted for model comparison, and the high R and R^2^ values indicate that all four models capture the overall trend of the MEX process well, with the predicted values closely scattered around the diagonal reference line. Because the printed-line width involves a relatively weaker nonlinear relationship than the edge non-uniformity, the four models generally achieve slightly better accuracy on the line width than on the edge non-uniformity. Among the four models, GPR delivers the most accurate predictions, which can be attributed to the high flexibility of its non-parametric formulation, whereas BPNN performs the least well under the limited dataset. MAE and RMSE were then introduced to further assess the four models, and the complete results based on the four classic evaluation indicators are summarized in Table 3 and Table 4. Consistent with the R and R^2^ rankings, the GPR model yields the lowest MAE and RMSE values on both the training and testing datasets, confirming it as the optimal model for predicting the printed-line features.

**Figure 12:**
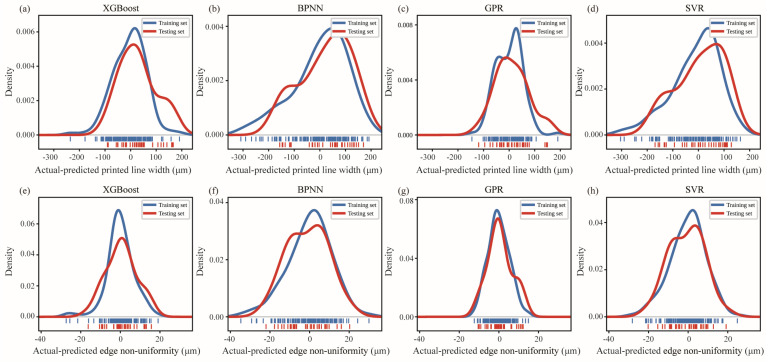
Kernel density estimation (KDE) distributions of the prediction residuals for the four models on the training and testing datasets: (**a**–**d**) line width and (**e**–**h**) edge non-uniformity.

Figure 12 presents the distribution and relative frequency of the residuals between the actual and predicted printed-line features. As shown in Figure 12a–d for the line width, the residuals of the GPR model are distributed within a relatively narrow band around zero, indicating a more concentrated error distribution than those of the XGBoost, BPNN, and SVR models, whose curves appear comparatively flatter and more widely spread. A similar pattern is observed for the edge non-uniformity in Figure 12e–h, where the GPR residuals are gathered more closely around zero, while the BPNN residuals exhibit a broader dispersion. For both responses, the training and testing curves of the GPR model remain in close agreement, which suggests reasonable generalization and limited overfitting. Overall, these results indicate that the GPR model tends to yield smaller and more concentrated prediction errors than the other three candidates, and it is accordingly regarded as the preferred model for predicting the printed-line features.

The predictive accuracy obtained here is consistent with, and in several respects exceeds, that reported for comparable machine learning studies of additive-manufacturing processes. Li et al. [25] compared multiple regressors for aerosol-jet printed-line prediction and likewise identified a kernel-based, Gaussian-process-type model as the most accurate, while Zhang et al. [28] reported R values around 0.95 for data-driven MEX quality modeling. Dejene and Lemu [39] and Reddy et al. [52] compared SVR, ANN, XGBoost, and other regressors for laser-powder-bed-fusion and micro-lattice surface-roughness prediction, reporting testing R^2^ values in the 0.80–0.92 range, comparable to the 0.86–0.91 achieved by the present GPR model, and they similarly found that ensemble and kernel methods outperform plain neural networks under small datasets. Sharma et al. [41] obtained R^2^ ≈ 0.90 for machine learning surface-roughness prediction, again in line with the present results. This agreement suggests that the proposed PSO-optimized GPR framework achieves competitive accuracy for MEX line-feature prediction, while also offering calibrated predictive uncertainty and SHAP-based interpretability, which have been less commonly addressed in previous studies.

### 4.3. Feature Analysis

**Figure 13:**
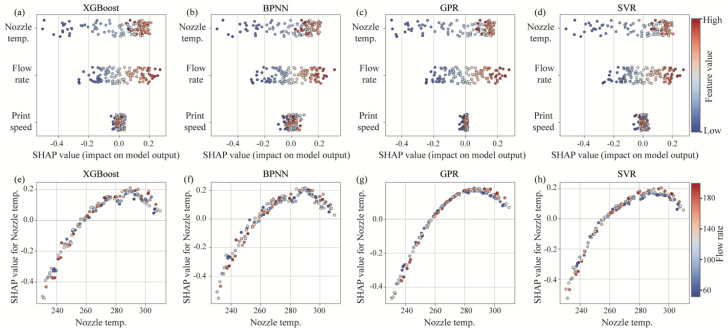
SHAP-based interpretability analysis for the printed-line width model: (**a**–**d**) beeswarm plots ranking feature importance and (**e**–**h**) dependence plots illustrating the effect of nozzle temperature.

As described by the SHAP method in Section 3.4, the Shapley values quantify the positive or negative contribution of each process parameter to the model output. Figure 13 presents the SHAP results for the line width model. As shown in Figure 13a–d, the parameters rank from top to bottom as nozzle temperature, flow rate, and print speed, indicating that the nozzle temperature most strongly affects the line width. A higher nozzle temperature and a larger flow rate both contribute positively to the line width, while the print speed plays a minor role. The dependence plots in Figure 13e–h further show that the nozzle temperature is positively correlated with the line width, with its effect rising rapidly at low temperatures and leveling off as the temperature increases.

**Figure 14:**
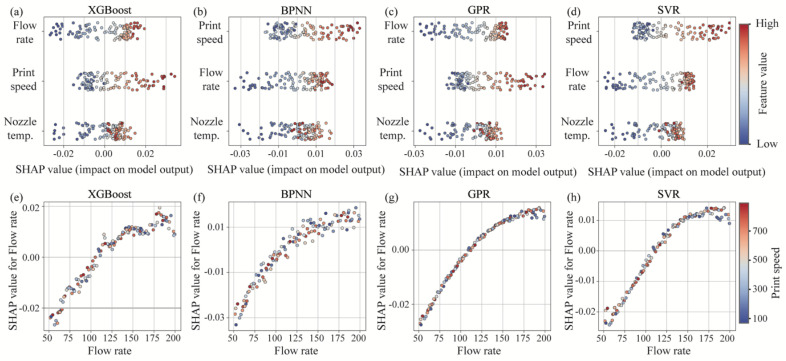
SHAP-based interpretability analysis for the printed-line edge non-uniformity model: (**a**–**d**) beeswarm plots ranking feature importance and (**e**–**h**) dependence plots illustrating the effect of flow rate.

Figure 14 presents the SHAP results for the edge non-uniformity model. As shown in Figure 14a–d, the parameters rank as flow rate, print speed, and nozzle temperature, indicating that the flow rate dominates the edge non-uniformity. Increasing either the flow rate or the print speed tends to raise the roughness. The dependence plots in Figure 14e–h show that the flow rate is positively correlated with the edge non-uniformity, with its effect increasing markedly at low flow rates and approaching saturation at high values, which suggests that an excessive flow rate noticeably increases the printed-line edge non-uniformity.

## 5. Conclusions

In this research, an integrated machine learning framework is proposed for the optimal prediction and analysis of printed-line features in material extrusion. Within the proposed framework, nozzle temperature, print speed, and flow rate were considered as input variables, while mean line width and edge non-uniformity were taken as the target responses. Four diverse machine learning algorithms (XGBoost, BPNN, GPR, and SVR) were adopted for model development. To enhance predictive accuracy, these models were optimized using Particle Swarm Optimization for automatic hyperparameter tuning. Through comparative evaluation of performance metrics, GPR was identified as the optimal model, consistently yielding the lowest error rates on both training and testing datasets. Additionally, a SHAP-based interpretability analysis quantified the underlying process dynamics, demonstrating that nozzle temperature primarily dictates the printed-line width, whereas the flow rate dominates the edge non-uniformity. By successfully capturing the nonlinear variability of track geometry within the working window, this interpretable and computationally efficient surrogate modeling framework provides a robust foundation for future inverse process design and closed-loop quality control.

Building on this foundation, future work will expand the dataset across multiple materials, nozzle geometries, and wider parameter ranges (aided by transfer learning), extend the framework to additional inputs and multiple quality responses through multi-output and physics-informed models, exploit the predictive variance of GPR for active-learning-based adaptive sampling, and ultimately, couple the interpretable surrogate with multi-objective optimization and in situ sensing to achieve experimentally validated, closed-loop process design and quality control.

## Figures and Tables

**Table 1:** Experimental setup of the MEX process.

Process Parameters			Experimental Conditions			
Print Speed	Flow Rate Multiplier	Nozzle Temperature	Material	Tip Diameter	Working Distance	Bed Temperature
60–900 mm/min	50–200%	230–310 °C	ABS	0.4 mm	0.2 mm	100 °C

**Table 2:** Main hyperparameters of each model to be optimized by PSO.

Model	Hyperparameter	Search Range
XGBoost	learning_rate	[0.01, 0.30] (log scale)
	n_estimators	[100, 1000] (integer)
	max_depth	[2, 8] (integer)
BPNN	alpha (L2)	[1 × 10^−6^, 1.0] (log scale)
	hidden_size_1	[8, 128] (integer)
	hidden_size_2	[0, 64] (integer; 0 = no 2nd layer)
GPR	length scale ℓ	[1 × 10^−2^, 1 × 10^2^] (log scale)
	noise variance σ_n_^2^	[1 × 10^−6^, 1.0] (log scale)
SVR	C	[1 × 10^−1^, 1 × 10^3^] (log scale)
	γ (gamma)	[1 × 10^−3^, 1 × 10^1^] (log scale)
	ε (epsilon)	[1 × 10^−4^, 1 × 10^−1^] (log scale)

**Table 3:** Modeling performance of printed-line width based on four classic evaluation indicators.

Model	Training Dataset				Testing Dataset			
	RMSE	MAE	R	R^2^	RMSE	MAE	R	R^2^
XGBoost	0.0644	0.0501	0.9670	0.9326	0.0761	0.0595	0.9464	0.8728
BPNN	0.1066	0.0845	0.9182	0.8151	0.0984	0.0852	0.8965	0.7869
GPR	0.0508	0.0421	0.9788	0.9580	0.0647	0.0516	0.9571	0.9080
SVR	0.0943	0.0733	0.9360	0.8553	0.0897	0.0757	0.9090	0.8230

**Table 4:** Modeling performance of printed-line edge non-uniformity based on four classic evaluation indicators.

Model	Training Dataset				Testing Dataset			
	RMSE	MAE	R	R^2^	RMSE	MAE	R	R^2^
XGBoost	0.0070	0.0050	0.9531	0.9008	0.0075	0.0059	0.9278	0.8596
BPNN	0.0111	0.0086	0.8823	0.7516	0.0101	0.0085	0.8705	0.7465
GPR	0.0053	0.0042	0.9713	0.9433	0.0060	0.0046	0.9549	0.9101
SVR	0.0090	0.0071	0.9273	0.8359	0.0087	0.0073	0.9059	0.8115

## Data Availability

The original contributions presented in this study are included in the article, and further inquiries can be directed to the corresponding author.
